# In-Situ Growth of Carbon Nanotubes on MOF-Derived High-Entropy Alloys with Efficient Electromagnetic Wave Absorption

**DOI:** 10.3390/ma19020239

**Published:** 2026-01-07

**Authors:** Zhongjing Wang, Bin Meng, Xingyu Ping, Qingqing Yang, Kang Wang, Shuo Wang

**Affiliations:** 1 Faculty of Materials Science & Engineering, Kunming University of Science & Technology, Kunming 650093, China20240209@kust.edu.cn (X.P.);; 2 Key Laboratory of Advanced Materials of Yunnan Province, Kunming University of Science & Technology, Kunming 650093, China; 3 Faculty of Information Engineering and Automation, Kunming University of Science & Technology, Kunming 650093, China; 4 Shanghai Aerospace Equipments Manufacture Co., Ltd., Shanghai 200245, China

**Keywords:** high-entropy alloy, metal-organic framework, carbon nanotubes, in-situ growth, electromagnetic wave absorption

## Abstract

To obtain an excellent electromagnetic wave (EMW) absorption material, a strategy was proposed in this study with the aid of in-situ growth of carbon nanotubes (CNTs) on the surface of a metal–organic framework (MOF)-derived FeCoNiMnMg high-entropy alloy (HEA). The HEA@CNT composite was successfully prepared via a solvothermal method combined with a one-step pyrolysis process. With the pyrolysis temperature increasing from 600 °C to 800 °C, the length of CNTs grew from 200 nm to about 600 nm approximately, while the defect density of CNTs was enhanced. This structural evolution significantly improved the dielectric properties and impedance matching. Consequently, the sample prepared at 800 °C (HEA@CNT-800) exhibited outstanding microwave absorption performances, achieving a minimum reflection loss (*RL_min_*) of −57.52 dB at a matched thickness of 2.3 mm and an effective absorption bandwidth (EAB) of 4.4 GHz at a thinner thickness of 1.9 mm. This work provides a novel perspective for designing high-performance MOF-derived absorption materials.

## 1. Introduction

As essential components for electromagnetic protection, stealth technology, and electronic device compatibility, electromagnetic wave (EMW) absorption materials perform a key role. Their mechanism of action lies in the efficient transformation of incident electromagnetic wave energy into dissipative thermal or other energy forms [1,2,3]. Ideal EMW absorption materials must satisfy two core requirements: (1) favorable impedance matching ensures sufficient penetration of electromagnetic waves into the material interior, which is a prerequisite for subsequent energy dissipation; (2) the material possesses pronounced loss capability, characterized by the efficient transformation of incident electromagnetic energy into heat or other energy forms for dissipation [4]. To date, researchers have developed various electromagnetic wave absorbing materials, such as ferrites [5,6,7] and carbon nanotubes [8,9,10,11], both of which can exhibit strong EMW absorption. However, the EMW absorption of ferrites relies solely on the magnetic loss mechanism, and that of carbon nanotubes depends only on the dielectric loss mechanism. If a material is primarily dominated by a single loss mechanism, it is often difficult to achieve a balance between the strong absorption (*RL_min_*) and broad bandwidth (EAB) at relatively thin matching thicknesses.

High-entropy alloys (HEAs) have attracted abundant attention as promising EMW absorbers, due to their distinctive high-entropy effect, lattice distortion effect, and “cocktail” effect [12,13]. A key advantage of high-entropy alloys (HEAs) over traditional materials arises from their multi-principal element composition. This characteristic allows for a broader scope in tailoring intrinsic electromagnetic parameters (complex permittivity and permeability), which is crucial for achieving ideal impedance matching and enhanced environmental adaptability [14]. For instance, Li et al. [15] reported in 2023 that FeCoNiCrCu exhibited good EMW absorption performances, with an *RL_min_* of −41.23 dB at 2.6 mm thickness and a maximum EAB of 4.5 GHz. However, bulk HEAs usually have a high density, while nano-sized HEA particles are prone to impedance mismatch due to the high electrical conductivity.

Simultaneously, metal–organic frameworks (MOFs), owing to their highly tunable structure and composition, have become ideal precursors for designing derived functional materials. Upon pyrolysis, MOFs can transform into porous carbon-based composite materials, where uniformly distributed metal nodes can be converted in-situ into magnetic nanoparticles, naturally forming a magnetic–dielectric composite structure [16]. Current research focus has shifted from single/bimetal MOFs [17,18,19,20,21] derivatives towards more complex trimetallic systems [22,23]. For example, Huang et al. [23] prepared ternary flower-like CoNiM@C(M=Cu, Zn, Fe, Mn) microspheres derived from Co-Ni-M-MOFs in 2024. Among them, the flower-like CoNiFe@C microspheres achieved an *RL_min_* value of −43.8 dB at 2.5 mm thickness, with a maximum EAB of 4.3 GHz at the same thickness. At a thickness of only 2.0 mm, the CoNiMn@C microspheres reached an *RL_min_* value of −30.1 dB at 14.2 GHz, and a maximum EAB of 5.8 GHz at 2.0 mm thickness. Therefore, increasing the number of metal components serves as an effective strategy to introduce abundant heterogeneous interfaces. This enhances interfacial polarization, which ultimately optimizes both impedance matching and loss capability synergistically. However, most studies on MOF-derived materials are confined to bi-/tri-metallic systems, while research on multi-metal high-entropy alloy systems is still in its infancy. Therefore, the introduction of multi-principal-element high-entropy alloys, leveraging their inherent severe lattice distortion and complex multi-element synergy, provides a broader design space for modulating the polarization behavior and impedance matching.

Currently, research aimed at performance breakthroughs now centers on designing multi-component composites for dielectric–magnetic synergy. By employing the in-situ growth of carbon nanotubes (CNTs) on magnetic components—a key microstructural design strategy—impedance matching can be optimized alongside the introduction of augmented loss mechanisms [24,25,26,27]. CNTs can construct a three-dimensional conductive network, enhancing conduction loss, while their inherent defects and interfaces with magnetic particles can significantly promote dipole polarization and interfacial polarization [28]. For instance, in 2025, Weng et al. [29] used nickel 1,3,5-benzenetricarboxylate (Ni-BTC) as a precursor to prepare MOF derivatives with in-situ grown CNTs on the surface, achieving *RL_min_* of −44.4 dB at a mere 1.72 mm thickness, laying the foundation for research on ultra-thin absorption materials. However, such in-situ CNT growth strategies are currently mostly applied to single or bimetallic MOF systems. Achieving controllable in-situ growth of CNTs on the surface of more complex multi-metallic MOF-derived high-entropy alloys and systematically studying their growth mechanism and electromagnetic response behavior has not been systematically reported and remains a significant challenge.

In this study, 1,3,5-benzenetricarboxylic acid is developed as the organic ligand and a solvothermal method is employed to prepare a multi-component MOF precursor. Carbon nanotubes are grown in-situ on its surface via chemical vapor deposition, synthesizing a MOF-derived high-entropy alloy@carbon nanotube (HEA@CNT) composite. The selection of the FeCoNiMnMg system in this study aims to construct a multifunctional high-entropy alloy substrate that combines synergistic magnetic and dielectric loss. The elements of Fe, Co, and Ni serve as primary magnetic components, ensuring substantial magnetic loss. Mn is incorporated to induce a pronounced lattice distortion due to its significant atomic size mismatch, thereby intensifying the electron scattering and potentially enhancing the polarization. Although Mg is non-magnetic, its large atomic radius difference maximizes the lattice strain effect. This research aims to investigate the regulation of the pyrolysis temperature on the morphology (length, defect density) of CNTs and reveals their growth mechanism on the HEA surface. At the same time, the influence mechanism of CNT morphological evolution on the electromagnetic parameters (complex permittivity and permeability) of the composites is analyzed systematically.

## 2. Experimental Procedure

### 2.1. Synthesis of HEF@CNT

Multi-metallic MOF precursor powders were synthesized using a one-step solvothermal method. Firstly, equimolar amounts of Co(NO_3_)_2_·6H_2_O, Ni(NO_3_)_2_·6H_2_O, Mn(NO_3_)_2_·4H_2_O, Mg(NO_3_)_2_·6H_2_O, and Fe(NO_3_)_3_·9H_2_O powders with a purity of 99.9% were dissolved in N,N-dimethylformamide (DMF). Subsequently, this metal salt solution was added dropwise into the DMF/ethanol (1:4) mixed solvent containing 1,3,5-benzenetricarboxylic acid (H_3_BTC), followed by stirring for 1 h. The mixture was transferred to a Teflon-lined autoclave and reacted at 130 °C for 48 h. After the reaction, the products were centrifugated, washed with DMF and ethanol, and finally dried at 60 °C to obtain the multi-metallic MOF precursor powders.

The HEA@CNT composite was prepared using a one-step pyrolysis method. The aforementioned MOF precursor and dicyandiamide (DCDA, as carbon source) were designed at a mass ratio of 1:5, and placed in the downstream and upstream zones of a tube furnace, respectively. Under an argon atmosphere, the temperature was raised to 400 °C at a rate of 5 °C/min and held for 2 h, then further increased to the target temperatures (600, 700, 800 °C) at a rate of 3 °C/min and maintained for 4 h. The resulting products were labeled as HEA@CNT-600, HEA@CNT-700, and HEA@CNT-800, respectively. For comparison, an HEA-800 sample was prepared at 800 °C without adding DCDA. All the above chemicals were purchased from Aladdin (London, UK) and Sinopharm Chemical Reagent Co., Ltd. (Shanghai, China).

### 2.2. Microstructure Characterization

To characterize the samples, several analytical techniques were utilized. The crystal structure was identified by X-ray diffraction (XRD, Cu Kα radiation, Bruker D8 Advance, Mannheim, Germany). Microstructural and morphological observations were carried out using scanning electron microscopy (SEM, JEOL JSM-7800F, JEOL, Tokyo, Japan) and transmission electron microscopy (TEM, JEOL JEM-1400; HRTEM, JEOL JEM-2100F, JEOL, Tokyo, Japan). X-ray photoelectron spectroscopy (XPS, Thermo Scientific K-Alpha, Thermo Scientific, Waltham, MA, USA) served to analyze the surface elemental composition and chemical states. Furthermore, Raman spectroscopy (Renishaw inVia, 532 nm laser, London, UK) was applied to investigate the graphitization degree and defects of the carbon nanotubes. XRD analysis was conducted in a continuous scan mode with a step of 0.02° and a speed of 2°/min. For SEM observation, the powders were directly adhered to conductive carbon tape. For TEM analysis, the powders of HEA@CNT-600, HEA@CNT-700, HEA@CNT-800 and HEA@C-800 were ultrasonically dispersed in ethanol and dropped onto carbon-coated copper grids.

### 2.3. Electromagnetic Properties

To evaluate the electromagnetic properties, we first prepared specimens by uniformly blending HEA@CNT powders with paraffin wax at a mass ratio of 4:6, followed by pressing them into coaxial toroids (3.0 mm inner diameter, 7.0 mm outer diameter). The complex permittivity (*ε_r_ = ε*′ *− jε*″) and permeability (*μ_r_ = μ*′ *− jμ*″) of these samples were then measured across 2–18 GHz via the coaxial line method employing an Agilent N5230A vector network analyzer (Agilent, Santa Clara, CA, USA). Finally, based on transmission line theory, the reflection loss (*RL*) and effective absorption bandwidth (EAB, frequency range with *RL* < −10 dB) were calculated; the specific formulas are provided in the Supporting Information. To ensure the reproducibility of the experimental data, the electromagnetic parameters for each composition (HEA@CNT-600/700/800) were measured twice to verify consistency.

## 3. Results and Discussion

### 3.1. Phase and Microstructure

**Figure 1:**
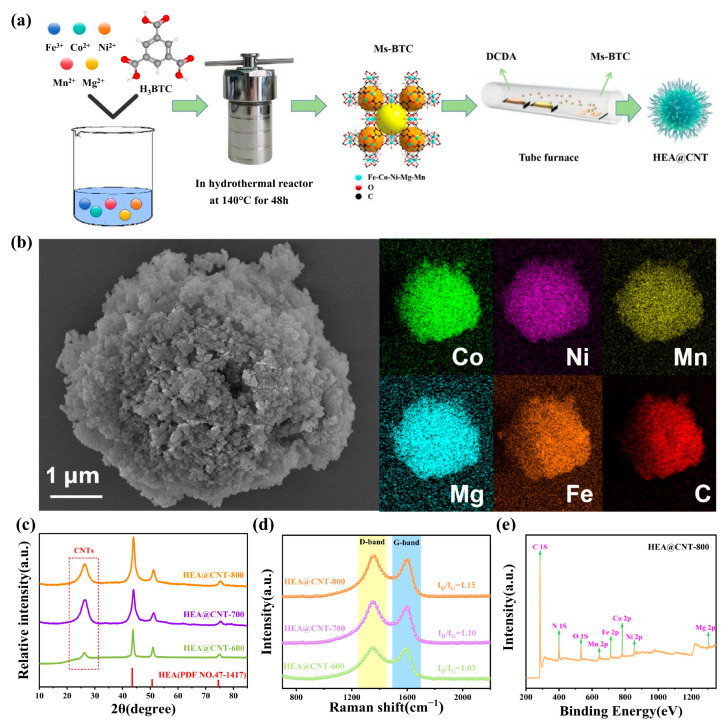
(**a**) Schematic illustration, (**b**) FE-SEM, EDS mapping, (**c**) XRD patterns, (**d**) Raman spectra and (**e**) XPS of HEA@CNT-600/700/800.

Figure 1a schematically illustrates the synthesis process of HEA@CNTs. During pyrolysis, the metal ions in the MOF precursor (Figure S1) are reduced and alloyed to form HEA nanoparticles, while CNTs grow in-situ on the surface of these HEA nanoparticles from the carbon-containing species decomposed from DCDA. SEM images (Figure 1b) show that the prepared multi-metallic MOF precursor exhibits three-dimensional flower-like microspheres assembled from nanosheets, with diameters ranging from approximately 3–8 μm. EDS elemental mapping shows the uniform distribution of the five metal elements (Co, Mn, Ni, Fe, Mg) within the MOF precursor (Figure 1b), indicating the successful synthesis of the multi-component MOF precursor. Figure 1c displays the XRD patterns of the HEA@CNT composites (HEA@CNT-600, HEA@CNT-700, and HEA@CNT-800). The dominant diffraction peaks for all samples can be readily indexed to a face-centered cubic (FCC) structure (space group: Fm-3m), confirming the successful formation of a single-phase FeCoNiMnMg high-entropy alloy solid solution. Additionally, a broad and low-intensity peak at approximately 26° is observed, which can be assigned to the (002) graphitic plane of the in-situ grown carbon nanotubes (CNTs). The weak and broad nature of this peak suggests that the CNTs possess a relatively low degree of graphitization and are potentially few-layered, which is consistent with the defective structure indicated by the Raman spectroscopy analysis (Figure 1d).

Figure 1d presents the Raman spectra of the HEA@CNT composites. All samples exhibit distinct characteristic peaks at approximately 1350 cm^−1^ and 1590 cm^−1^, corresponding to the D band and G band of carbon materials, respectively. The D band is associated with structural disorders in the carbon atomic lattice, such as sp^3^-hybridized defects, vacancies, and grain boundaries. In contrast, the G band originates from the in-plane vibration of sp^2^-hybridized carbon atoms in hexagonal rings, serving as a marker for graphitic ordering [30]. As the pyrolysis temperature rises from 600 °C to 800 °C, the *I_D_*/*I_G_* intensity ratio of the materials systematically increases from 1.04 to 1.15. This trend indicates that although higher temperatures facilitate carbon graphitization, the rapid growth of CNTs in this process simultaneously introduces a higher density of structural defects. To further quantify the defect evolution, the average defect distance (*L_D_*) and defect density (*n_D_*) of the sp^2^ carbon clusters within the CNTs are calculated based on the Raman spectral data (see Supporting Information for equations). The results demonstrate that *L*_D_ gradually decreases, while *n*_D_ increases significantly with the rise in pyrolysis temperature. This quantitatively confirms that the longer CNTs formed at 800 °C possess a high concentration of topological defects (e.g., pentagon/heptagon pairs causing tube wall curvature) and atomic vacancy defects [31]. These defects play a crucial role in electromagnetic wave absorption: atomic vacancies and heteroatom defects can act as electric dipoles, inducing significant dipole polarization relaxation under the alternating electromagnetic field [32]. Meanwhile, the abundant grain boundaries and distorted carbon layer structures can localize numerous charge carriers, enhancing conductive loss and interface polarization. Therefore, the increased pyrolysis temperature not only promotes CNT growth but also concurrently elevates their defect density, introducing more polarization centers and loss pathways into the composite, thereby synergistically enhancing the electromagnetic wave absorption performance.

The abundant defects within the material act as polarization centers, which localize bound electrons and induce dipole polarization, thereby significantly enhancing electromagnetic wave absorption. This polarization mechanism is corroborated by the XPS analysis of HEA@CNT-800 (Figure 1e and Figure S2). The spectra indicate that all metallic elements coexist in metallic and oxidized states: the metallic state originates from the high-entropy alloy core, while the oxidized state results from surface oxidation of the nanoparticles due to their high surface energy. The presence of oxides on the HEA surface is typically beneficial for the electromagnetic wave absorption in metal-based composite materials, primarily by enhancing the interface polarization loss. The formation of surface oxides can generate abundant heterogeneous interfaces (e.g., HEA-core/oxide, oxide/CNT). The interfacial polarization and relaxation induced at these interfaces serve as important dielectric loss mechanisms.

**Figure 2:**
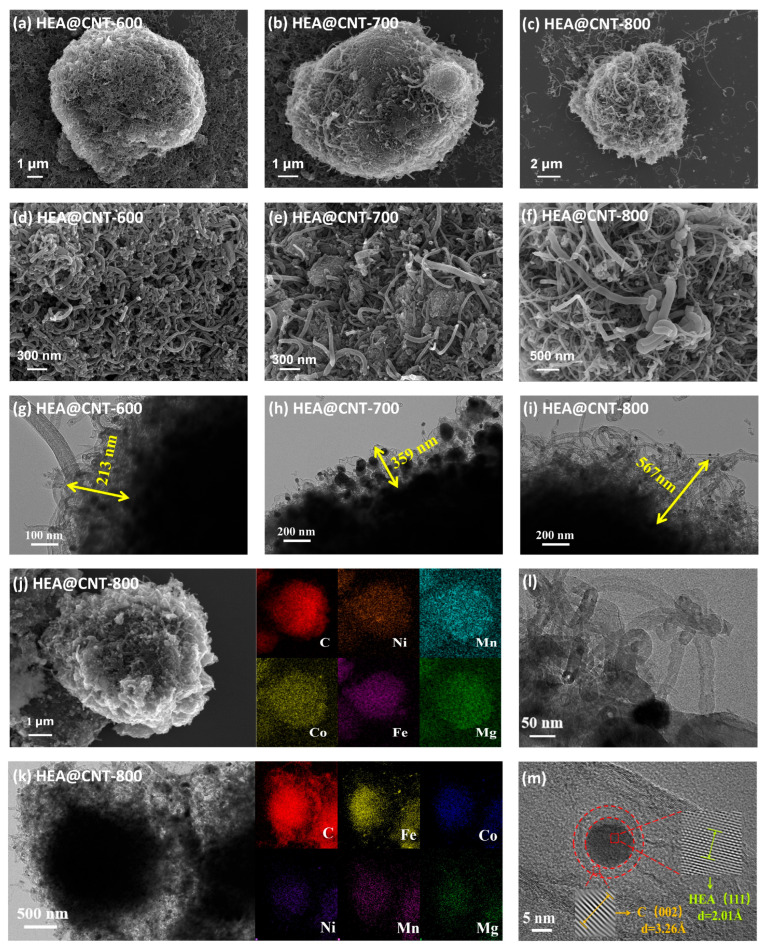
(**a**–**f**) SEM images of HEA@CNT-600/700/800, (**g**–**i**) TEM images of HEA@CNT-600/700/800, (**j**,**k**) EDS elemental mapping of HEA@CNT-800, (**l**,**m**) HR-TEM images of selected areas.

The morphologies of HEA@CNT are further characterized by SEM and TEM (Figure 2a–i). After heat treatment and pyrolysis, the surface of HEA-800 (prepared without DCDA) becomes rougher, but no CNT formation can be observed (Figure S3a). XRD analysis confirms that the primary phase composition of HEA-800 is solely the alloy phase (Figure S3b). In contrast, CNTs successfully grow in all HEA@CNT samples (Figure 2a–f), confirming the effectiveness of DCDA as a carbon source. EDS elemental mapping (Figure 2j) shows an equimolar ratio (Figure S4) and uniform distribution of the five metal elements, further evidencing the formation of the high-entropy alloy. TEM images (Figure 2g–i) clearly reveal the microstructure of the CNTs. Increasing the pyrolysis temperature from 600 °C to 800 °C promotes substantial growth in CNT length, as evidenced by statistical measurements showing an increase from 200 nm to 600 nm. In HEA@CNT-800 (Figure 2l), typical bamboo-like CNTs with diameters of approximately 50 nm can be observed. High-resolution TEM (Figure 2m) and the corresponding EDS elemental mapping (Figure 2j) show that HEA nanoparticles are encapsulated at the tips or embedded within the CNTs, confirming that the CNTs follow a tip-growth mode [29,33]. This growth mode stems from the catalytic cracking of pyrolytic carbon by the HEA nanoparticles.

### 3.2. Electromagnetic Wave Absorption Performances

**Figure 3:**
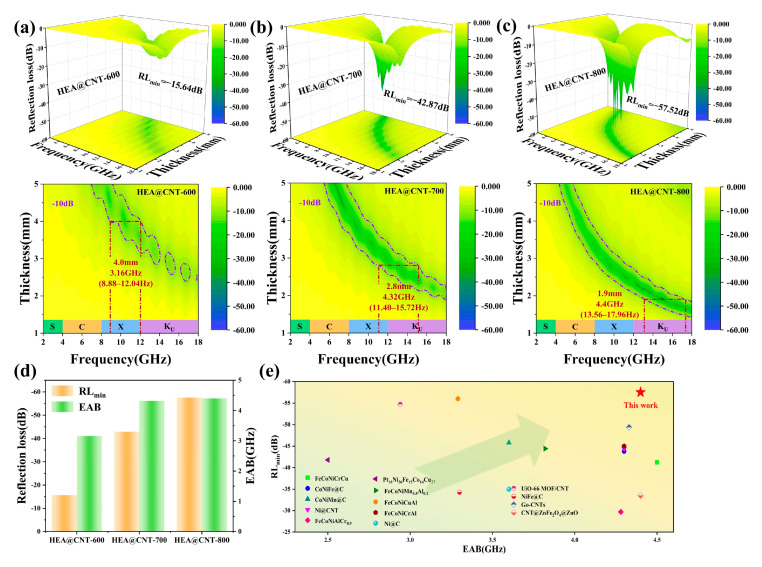
Reflection loss (*RL*) and corresponding 2D maps of samples (**a**) HEA@CNT-600, (**b**) HEA@CNT-700 and (**c**) HEA@CNT-800, (**d**) The RL_min_ and *EAB_max_* values of HEA@C-600/700/800, (**e**) *RL_min_* and *EAB_max_* of the present work and other wave-absorbing materials.

From the established electromagnetic parameters, the reflection loss (*RL*) and effective absorption bandwidth (EAB, *RL* < −10 dB) of the HEA@CNT composites across different thicknesses were determined, as summarized in Figure 3. Among them, HEA@CNT-800 demonstrates the best performance (Figure 3c). At an optimal matching thickness of 2.3 mm, it achieves a minimum reflection loss (*RL_min_*) of −57.52 dB at 12.44 GHz. At a thickness of 1.9 mm, its EAB reaches 4.4 GHz (covering 13.56–17.96 GHz), almost encompassing the entire Ku-band. In comparison, the best *RL_min_* values for HEA@CNT-600 and HEA@CNT-700 are −35.2 dB and −42.87 dB, with corresponding EAB values of 3.16 GHz (at 4.0 mm) and 4.32 GHz (at 2.8 mm), respectively. Figure 3d summarizes the *RL_min_* and maximum EAB for the three samples. Figure 3e and Table S1 compares the performance of HEA@CNT-800 with other recently reported EMA materials, indicating that HEA@CNT prepared in this work possesses competitive performance in terms of comprehensive performances characterized by “thin thickness, strong absorption, and wide bandwidth”.

### 3.3. Electromagnetic Wave Absorption Mechanism

**Figure 4:**
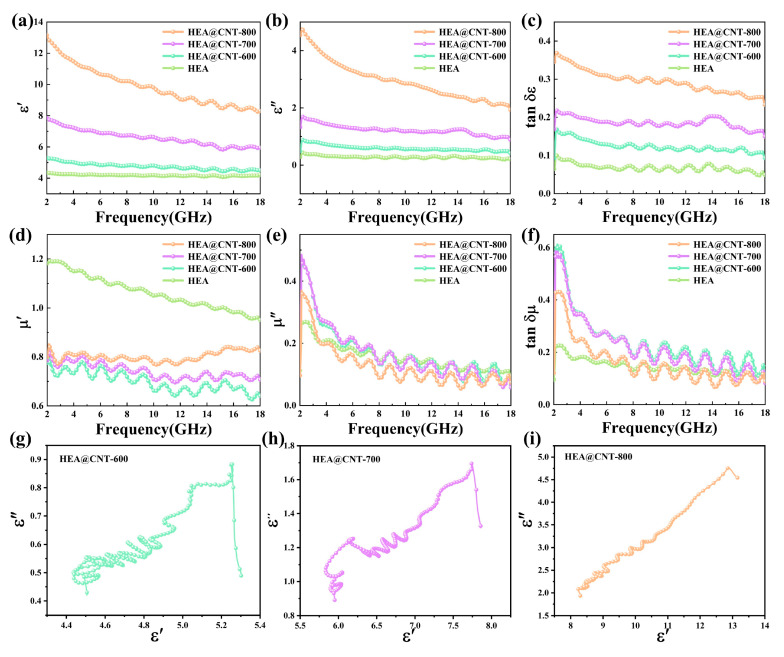
(**a**) *ε*′, (**b**) *ε*″, (**c**) tan *δε*, (**d**) *μ*′, (**e**) *μ*″, (**f**) tan *δμ*, Cole–Cole plot of (**g**) HEA@CNT-600, (**h**) HEA@CNT-700 and (**i**) HEA@CNT-800.

To gain deeper insight into the performance enhancement mechanism, the electromagnetic parameters of the HEA@CNT are analyzed. Figure 4a–c display the dielectric properties of the composites. Generally, the real part of the complex permittivity (*ε*′) represents the ability to store electrical energy, while the imaginary part (*ε*″) signifies the capability to dissipate energy in an alternating electromagnetic field [34,35,36]. Both *ε*′ and *ε*″ values decrease with the test frequency, indicating a typical dispersion behavior associated with loss characteristics [37,38]. Both the real part (*ε*′) and imaginary part (*ε*″) of the complex permittivity increase significantly with the rising pyrolysis temperature (Figure 4a,b), primarily attributed to the enhanced conductive network formed by CNTs and the increased defect density.

The dielectric loss tangent (*tan δε* = *ε*″/*ε*′) also follows the same trend (Figure 4c). Furthermore, across the entire frequency band, *tan δε* is greater than the magnetic loss tangent (*tan δμ* = *μ*″/*μ*′) (Figure 4c,f), indicating that dielectric loss is the predominant loss mechanism in HEA@CNT [39]. The real part (*μ*′) and imaginary part (*μ*″) of the complex permeability show minor variations among the different samples (Figure 4d,e), suggesting that the heat treatment temperature primarily modulates the dielectric properties rather than the magnetic properties. The *μ*″ curves exhibit several broadened resonance peaks within the 2–18 GHz range, indicating the presence of multiple magnetic loss mechanisms such as natural resonance and exchange resonance [40]. The calculation of the eddy current loss coefficient *C*_0_ (*C*_0_ = *µ*″(*µ*′)^−2^*f*^−1^) reveals that its value is not constant (Figure S5), confirming that eddy current loss is not the sole source of magnetic loss [41,42]. The variation of *C*_0_ with frequency suggests that the multiple mechanisms, including natural resonance and exchange resonance, collectively contribute to the magnetic loss.

Multiple fluctuation peaks in the mid-to-high frequency region of the *ε*″ curves correspond to multiple relaxation polarizations. This behavior, induced by the synergistic combination of HEA and CNTs, constitutes a key EMW loss mechanism, further confirming the material’s effective design. The significant *ε*″ values align with free electron theory, which correlates *ε*″ with the material’s electrical conductivity [43,44,45,46,47]. The dielectric relaxation behavior can be effectively interpreted using the Debye model [48,49,50,51]. The presence of multiple distinct semicircles in the Cole–Cole plots (Figure 4g–i) signifies the contribution of several relaxation processes within the HEA@CNT composites. Each semicircle is indicative of a specific polarization mechanism, with the number of observed arcs correlating with the diversity of polarized dipoles. Furthermore, the conduction loss, which originates from the migration of charge carriers within the conductive network, is identified as the primary source of the pronounced tail-like features observed at lower frequencies. As shown in Figure 4g–i, the Cole–Cole plots for all HEA@CNT samples consist of multiple distorted semicircles and linear tails. The multiple semicircles suggest the existence of multiple relaxation processes, originating from defect-induced dipole polarization within the CNTs themselves and interfacial polarization induced by the heterogeneous interfaces between the HEA and CNTs. The linear tails are associated with the conduction loss generated by the CNT/HEA conductive network, and HEA@CNT-800 exhibits the longest tail, indicating its strongest conduction loss.

**Figure 5:**
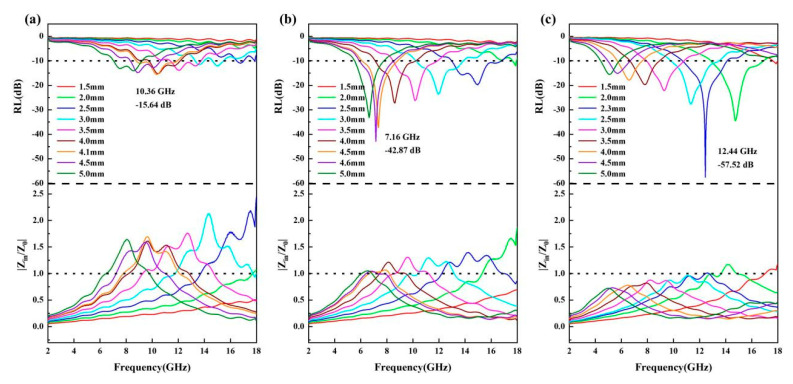
2D RL and impedance matching of (**a**) HEA@CNT-600, (**b**) HEA@CNT-700 and (**c**) HEA@CNT-800.

Furthermore, the analysis of EMW absorption performance variation hinges on two principles [52,53]. The attenuation constant (*α*) characterizes the material’s ability to dissipate electromagnetic energy, whereas the impedance matching parameter *Z* (*Z = |Z_in_*/*Z*_0_*|*) describes the efficacy with which waves can enter the absorber. Together, they reveal the underlying reasons for performance differences. The value of *α* can be calculated according to Equation (S9) (Figure S6). As shown in Figure S6, the HEA@CNT-800 composite exhibits the largest attenuation constant compared to HEA@CNT-600 and HEA@CNT-700. For impedance matching, the value of Z can be calculated by:

$$Z=\left|\frac{Z_{\mathrm{in}}}{Z_{0}}\right|=\sqrt{\frac{\mu_{r}}{\varepsilon_{r}}}\tanh\left[j\left(\frac{2f\pi d}{c}\right)\sqrt{\mu_{r}\varepsilon_{r}}\right]$$

where *ε_r_* and *μ_r_* are the relative complex permittivity and permeability of the composite medium, respectively, *Z_in_* is the normalized input impedance, *c* is the speed of light in vacuum, f is the frequency of the electromagnetic wave, j is an imaginary unit, and *d* is the absorption layer thickness. A *Z* value closer to 1 is considered indicative of ideal impedance matching performance. The condition of zero reflection, where electromagnetic waves enter the material without impedance, is defined by an ideal *Z* value of 1.0. Good impedance matching coupled with strong loss capability leads to excellent microwave absorption performance. As depicted in Figure 5, by plotting the frequency distribution of *|Z_in_*/*Z*_0_*|* at different thicknesses, it was found that for HEA@CNT-800, near its optimal absorption thickness (2.3 mm), the *|Z_in_*/*Z*_0_*|* value almost approaches 1 over a wider frequency range (Figure 5c), indicating the achievement of the best impedance matching. The high defect density in CNTs provides abundant dipoles for polarization loss. Simultaneously, the complex HEA/CNT interface serves as a major site for strong interfacial polarization. These two factors are inherently interrelated, as defects can also localize charges near the interface, thereby amplifying the polarization effect. At the same time, the longer CNTs form a more effective three-dimensional conductive network. The unique dielectric properties resulting from the combined effects described above (enhanced *ε*″ from polarization and modulated *ε*′ from interfaces) achieve an optimal balance with the magnetic properties of the HEA. This balance is reflected in the calculated impedance-matching parameter (|*Z_in_*/*Z*_0_|) being close to 1 over a broad frequency range for HEA@CNT-800. Therefore, the favorable impedance matching is not an independent characteristic, but rather a direct result of the optimized dielectric properties achieved through defect and interface engineering.

The coexistence of multiple metal atoms (FeCoNiMnMg) within the same solid-solution lattice of the high-entropy alloy (HEA) leads to significant lattice distortion and a complex surface chemical environment. Experimental data show that with the rise in pyrolysis temperature, the HEA promotes the growth of longer CNTs (length increasing from 200 nm to 600 nm) and introduces a higher defect density (Raman *I_D_*/*I_G_* ratio rising from 1.04 to 1.15). These structural changes directly enhance the dielectric loss capability of the composite. Meanwhile, the coexistence of multiple metallic elements and their surface environment results in more complex electromagnetic characteristics at the HEA/CNT interface, which helps to regulate the overall permittivity and permeability of the material, thereby achieving improved impedance matching (|*Z_in_*/*Z*_0_| close to 1 at a thickness of 2.3 mm). Compared with traditional catalysts containing only one or two metals, this multi-component HEA system offers higher possibilities for the synergistic optimization of “structural growth, defect modulation, and impedance matching”, providing a new design strategy for high-performance absorbing materials.

## 4. Conclusions

This study successfully demonstrated the in-situ growth of carbon nanotubes (CNTs) on a MOF-derived FeCoNiMnMg high-entropy alloy (HEA) within a pyrolysis temperature range of 600–800 °C, achieved through a combination of solvothermal and chemical vapor deposition methods. The systematic investigation revealed a significant influence of pyrolysis temperature on the microstructure and properties of the resulting HEA@CNT composites. Elevating the pyrolysis temperature from 600 °C to 800 °C induced a substantial expansion in CNT length from approximately 200 nm to about 600 nm. Concurrently, the Raman *I_D_*/*I_G_* intensity ratio rose from 1.04 to 1.15, indicating a corresponding growth in defect density. These controlled structural modifications directly resulted in a systematic evolution of the composites’ electromagnetic parameters: the real part of the complex permittivity (*ε*′) was elevated from 4.4–5.3 to 8.2–13.1, while the imaginary part (*ε*″) demonstrated a substantial enhancement from 0.42–0.88 to 1.93–4.78. This confirms that the development of an extended CNT network, coupled with a higher defect density, synergistically augmented both conduction loss and polarization relaxation. The exceptional electromagnetic wave absorption performance is attributed to the synergistic combination of optimized impedance matching (with HEA@CNT-800 exhibiting a |*Z_in_*/*Z*_0_| value closest to 1) and intensified dielectric and interfacial polarization losses. Consequently, the HEA@CNT-800 composite synthesized at 800 °C delivered optimal performance, achieving a minimum reflection loss of −57.52 dB at a matched thickness of 2.3 mm and an effective absorption bandwidth of 4.4 GHz at a thickness of 1.9 mm. This work establishes the construction of CNT architectures on MOF-derived high-entropy alloys as a viable strategy toward designing high-performance, MOF-based microwave absorption materials. Although this study demonstrates the potential of MOF-derived HEA as an advanced substrate for CNT growth and high-performance absorption, future studies will further explore the vast compositional space of HEAs (e.g., varying the ratio of magnetic to non-magnetic elements) to further decouple and optimize catalytic, magnetic, and interfacial properties.

## Data Availability

The original contributions presented in this study are included in the article/Supplementary Materials. Further inquiries can be directed to the corresponding author.

## Supplementary Materials

The following supporting information can be downloaded at: https://www.mdpi.com/article/10.3390/ma19020239/s1, Figure S1: XRD patterns of HEA@CNT precursor; Figure S2: XPS spectra of Co 2p, C1s, Fe 2p, Mg 2p, Mn 2p and Ni 2p of HEA@CNT-800; Figure S3: (a) SEM and (b) XRD patterns of HEA-800; Figure S4: EDS quantitative analysis results of HEA@CNT-800; Figure S5: Frequency dependence of the eddy current loss coefficient (C_0_) for HEA@CNT composites; Figure S6: Frequency dependence of the attenuation constant (α) for HEA@CNT composites; Figure S7: CNT length distribution of (a) HEA@CNT-600, (b) HEA@CNT-700 and (c) HEA@CNT-800; Table S1: Microwave absorption properties of HEA@CNT-800 and other wave absorbing materials. References [15,23,27,29,54,55,56,57,58,59,60,61,62,63] are cited in the Supplementary Materials.
