# Supplementary material for: In-Situ Growth of Carbon Nanotubes on MOF-Derived High-Entropy Alloys with Efficient Electromagnetic Wave Absorption
Source: Materials (Basel). 2026 Jan 7;19(2):239. doi: 10.3390/ma19020239 (PMC12843484; doi:10.3390/ma19020239)
Supplement: Supplementary file 1 [file materials-19-00239-s001.zip › materials-4064880-supplementary.pdf]

Supplementary Materials

In-Situ Growth of Carbon Nanotubes on MOF-Derived High-Entropy Alloys with Efficient Electromagnetic Wave Absorption

Zhongjing Wang [1,2], Bin Meng [1,2,*], Xingyu Ping [1,2], Qingqing Yang [3], Kang Wang [4] and Shuo Wang [1,2]

[1]Faculty of Materials Science & Engineering, Kunming University of Science & Technology, Kunming 650093, China

[2]Key Laboratory of Advanced Materials of Yunnan Province, Kunming University of Science & Technology, Kunming 650093, China

[3]Faculty of Information Engineering and Automation, Kunming University of Science & Technology, Kunming 650093, China

[4]Shanghai Aerospace Equipments Manufacture Co., Ltd., Shanghai 200245, China

[*]Corresponding Author: Bin Meng, Kunming University of Science & Technology, P. R. China; E-mail: mengbin@kust.edu.cn; Tel: 86-871-65109952

Electromagnetic wave absorption experiments

Firstly, the absorbing agents were heated and mixed with paraffin according to different mass percentages to form a standard ring shape (outer diameter of 7.00 mm, inner diameter of 3.04 mm). Afterwards, the electromagnetic parameters of the absorbing agents were measured using a vector network analyzer. Lastly, the electromagnetic parameters were used to calculate the electromagnetic loss and the absorption bandwidth according to the transmission line theory equations S3 and S4.

Data analysis

(1) The average distance between defects (L_D):

$$L_D(\text{nm})=\sqrt{(1.8\pm0.5)\times10^{-9}\times\lambda_L^4\times\left(\frac{I_D}{I_G}\right)^{-1}} \quad \text{(Equation S1)}$$

(2) The Defect density (n_D):

$$n_D=\frac{10^{14}}{\pi L_D^2} \quad \text{(Equation S2)}$$

$$n_D(\text{cm}^{-2})=(1.85\pm0.5)\times10^{22}\times\lambda_L^{-4}\times\left(\frac{I_D}{I_G}\right)$$

Where λ_L is the wavelength of excitation light, its value is 532 nm.

(3) According to the transmission line theory, the reflection loss (*RL*) value of samples can be calculated from the measured electromagnetic parameters.

$$RL(\text{dB})=20\log\left|\frac{Z_{in}-Z_0}{Z_{in}+Z_0}\right| \quad \text{(Equation S3)}$$

$$Z_{in}=Z_0\sqrt{\frac{\mu_r}{\varepsilon_r}}\tanh\left[j\left(\frac{2\pi fd}{c}\right)\sqrt{\mu_r\varepsilon_r}\right] \quad \text{(Equation S4)}$$

Where ε_r is the negative permittivity of the absorbing material, μ_r is its complex permeability, c is the speed of light in a vacuum, f is the frequency of the electromagnetic wave, d is the thickness of the wave-absorbing material used and j is an imaginary unit.

RL value lower than −10 dB is usually needed for electromagnetic wave absorption materials, which means that more than 90% of electromagnetic waves could be

absorbed. The electromagnetic wave frequency range with the *RL* values lower than −10 dB is defined as effective absorption bandwidth (EAB).

(4) According to the Debye relaxation theory, the real (ε') and imaginary (ε'') parts of the complex permittivity of a material can be defined by the following equations:

$$\varepsilon' = \varepsilon_\infty + \frac{(\varepsilon_s - \varepsilon_\infty)}{1+\omega^2 t^2} \quad \text{(Equation S5)}$$

$$\varepsilon'' = \frac{(\varepsilon_s - \varepsilon_\infty)\omega t}{1+\omega^2 t^2} \quad \text{(Equation S6)}$$

(5) The relative magnetic permeability (μ_r) can be calculated from the following equation S7:

$$\mu_r = \frac{M_s}{\mu_0 (akH_cM_s + b\lambda\xi)} \quad \text{(Equation S7)}$$

Where a and b are the material-dependent constants, and λ, k, and ξ are the magnetostriction coefficient, proportional coefficient, and internal strain, respectively.

(6) The impedance matching is evaluated by $|Z_{in}/Z_0|$ according to the equations:

$$Z_0 = \sqrt{\frac{\mu_0}{\varepsilon_0}} \quad \text{(Equation S8)}$$

Where μ_0 and ε_0 are the permittivity and permeability of free space, respectively.

(7) The attenuation coefficient (α) equation S10:

$$\alpha = \frac{\sqrt{2}\pi f}{c}\sqrt{(\mu''\varepsilon''-\mu'\varepsilon')+\sqrt{(\mu''\varepsilon''-\mu'\varepsilon')^2+(\mu'\varepsilon''+\mu''\varepsilon')^2}} \quad \text{(Equation S9)}$$

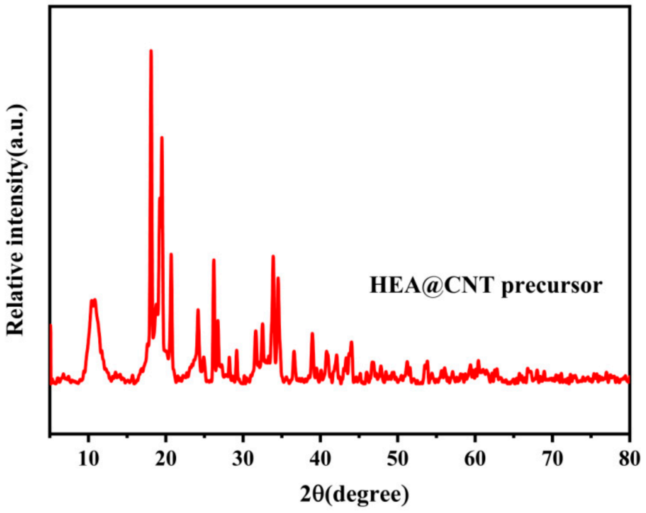

Figure S1 XRD patterns of HEA@CNT precursor

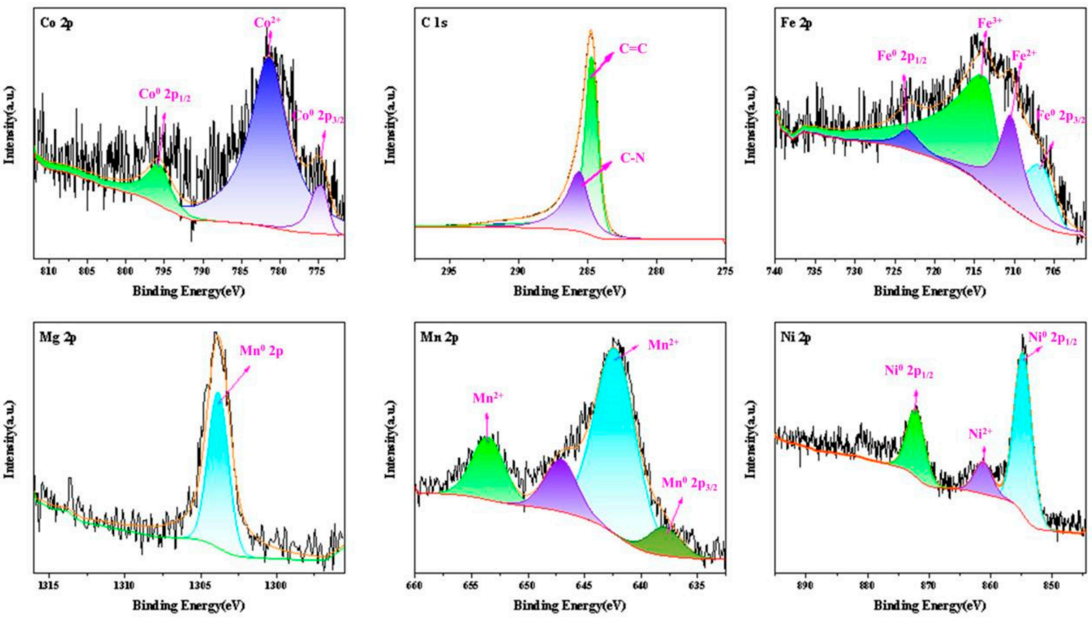

Figure S2 XPS spectra of Co 2p,C1s, Fe 2p,Mg 2p, Mn 2p and Ni 2p of HEA@CNT-800

(a)

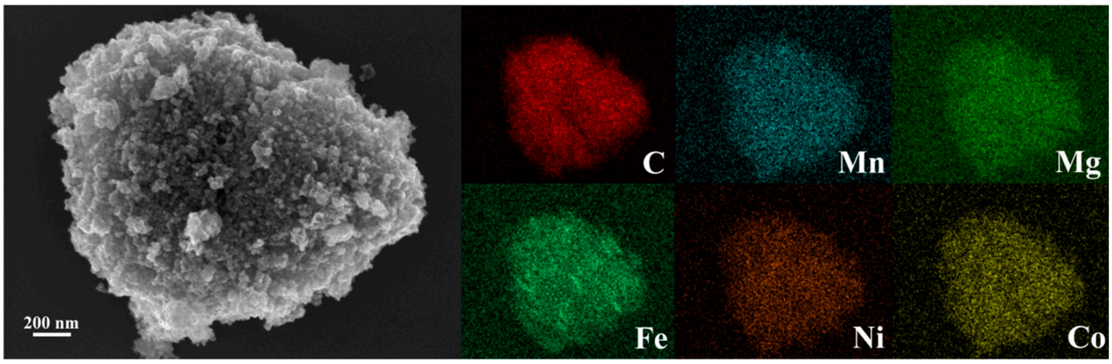

(b)

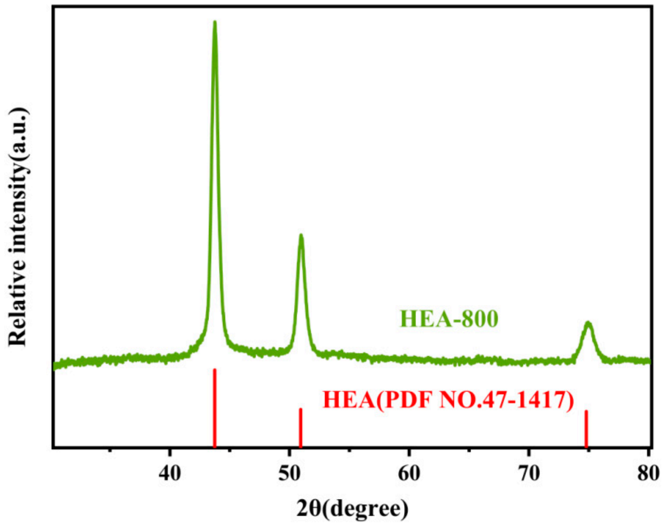

Figure S3 (a) SEM and (b) XRD patterns of HEA-800

.

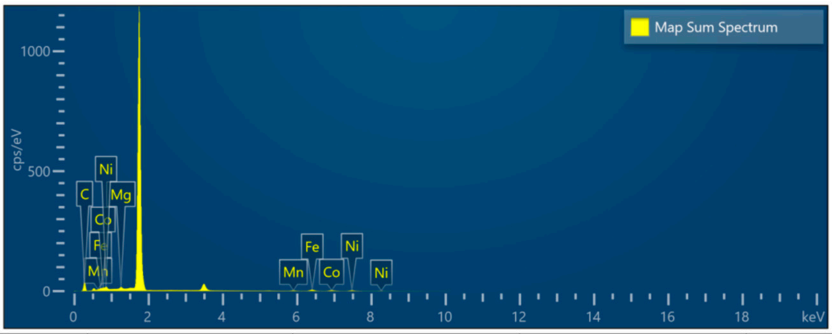

Map Sum Spectrum				
Element	Line Type	Weight %	Weight % Sigma	Atomic %
C	K series	61.52	0.23	87.45
Mg	K series	2.75	0.06	1.93
Mn	K series	3.36	0.10	1.04
Fe	K series	11.70	0.14	3.58
Co	K series	9.43	0.16	2.73
Ni	K series	11.24	0.18	3.27
Total		100.00		100.00

Figure S4 EDS quantitative analysis results of HEA@CNT-800

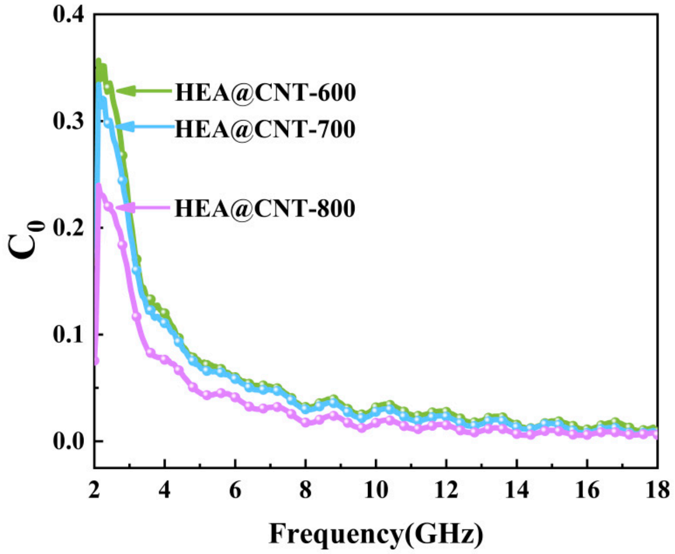

Figure S5 Frequency dependence of the eddy current loss coefficient (C_0) for HEA@CNT composites

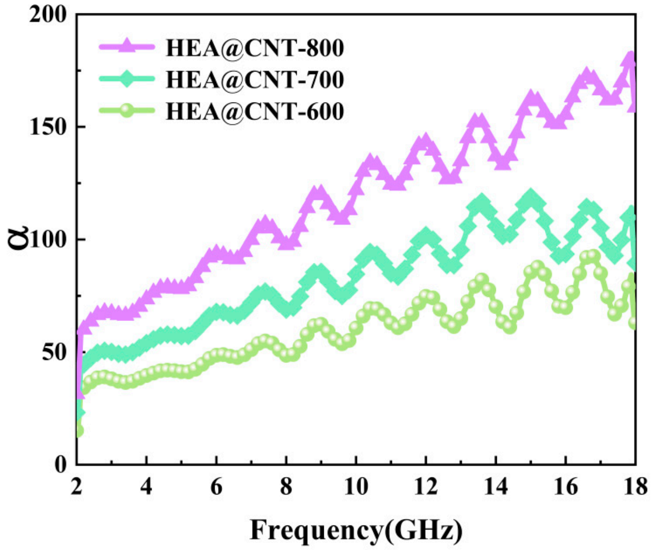

Figure S6 Frequency dependence of the attenuation constant (α) for HEA@CNT composites

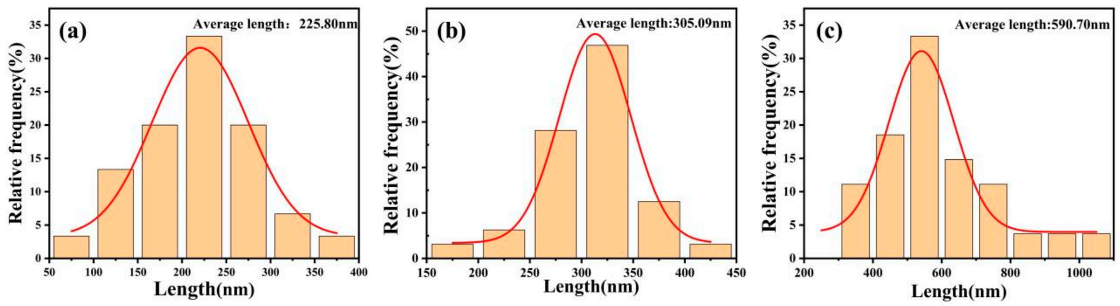

Figure S7 CNT length distribution of (a)HEA@CNT-600, (b)HEA@CNT-700 and (c)HEA@CNT-800

Table S1 Microwave absorption properties of HEA@CNT-800 and other wave absorbing materials

Sample	RLmin (dB)	EABmax (GHz)	Thickness (mm)	Filling (wt%)	Reference
FeCoNiCuCr	-41.23	4.50	4.00	80	**[1]**
CoNiFe@C	-43.8	4.3	2.5	60	**[2]**
$(Mo_{0.25}Cr_{0.25}Ti_{0.25}V_{0.25})_3AlC_2$	-45.80	3.60	1.70	75	**[3]**
Ni@CNT	-44.4	4.3	1.72	70	**[4]**
$FeCoNiAlCr_{0.9}$	-29.72	4.28	2.00	50	**[5]**
$Pt_{18}Ni_{26}Fe_{15}Co_{14}Cu_{27}$	-41.80	2.50	4.00	/	**[6]**
$FeCoNiMn_{0.5}Al_{0.2}$	-44.425	3.82	3.50	60	**[7]**
FeCoNiCuAl	-56.04	3.29	3.23	65	**[8]**
FeCoNiCrAl	-45	4.3	1.5	/	**[9]**
Ni@C	-52.9	4.6	2	40	**[10]**
UiO-66 MOF/CNT	-54.74	2.94	2.04	10	**[11]**
NiFe@C	-34.29	3.3	2.3	30	**[12]**
Go-CNTs	-49.4	4.33	2.2	65	**[13]**
CNT@$ZnFe_2O_4$@ZnO	-33.5	4.4	/	40	**[14]**
HEA@CNT-800	-57.52	4.4	1.9	40	**This work**

Reference

[1] G. Li, H. Zhao, H. Wang, Z. Zhou, L. Gao, W. Su, C. Dong, Enhanced microwave absorption performances of FeCoNiCuCr high entropy alloy by optimizing particle size dehomogenization, J. Alloys Compd. 941 (2023) 168822.

[2] M. Huang, B. Li, Y. Qian, L. Wang, H. Zhang, C. Yang, L. Rao, G. Zhou, C. Liang, R. Che, MOFs-Derived Strategy and Ternary Alloys Regulation in Flower-Like Magnetic-Carbon Microspheres with Broadband Electromagnetic Wave Absorption, Nano-Micro Lett. 16 (2024) 245.

[3] L. Qiao, J. Bi, G. Liang, C. Liu, Z. Yin, Y. Yang, H. Wang, S. Wang, M. Shang, W. Wang, Synthesis and electromagnetic wave absorption performances of a novel $(Mo_{0.25}Cr_{0.25}Ti_{0.25}V_{0.25})_3AlC_2$ high-entropy MAX phase, J. Mater. Sci. Technol. 137 (2023) 112–122.

[4] J. Weng, Y. Liu, X. Huang, Synthesis of in situ grown CNTs on MOF-derived Ni@CNT with tailorable microstructures toward regulation of electromagnetic wave absorption performance, Carbon 231 (2025) 119678.

[5] Y. Duan, H. Pang, X. Wen, X. Zhang, T. Wang, Microwave absorption performance of $FeCoNiAlCr_{0.9}$ alloy powders by adjusting the amount of process control agent, J. Mater. Sci. Technol. 77 (2021) 209–216.

[6] P. Wu, X. Kong, Y. Feng, W. Ding, Z. Sheng, Q. Liu, G. Ji, Phase Engineering on Amorphous/Crystalline γ-Fe_2O_3 Nanosheets for Boosting Dielectric Loss and High-Performance Microwave Absorption, (n.d.).

[7] H. Pang, Y. Duan, M. Gao, L. Huang, X. Liu, Z. Li, Electromagnetic wave absorption performance of $FeCoNiMn_{0.5}Al_{0.2}$ high entropy alloys governed by nanocrystal evolution, Mater. Today Nano 20 (2022) 100243.

[8] Y. Zhan, L. Xia, H. Yang, N. Zhou, G. Ma, T. Zhang, X. Huang, L. Xiong, C. Qin, W. Guangwu, Tunable electromagnetic wave absorbing properties of carbon nanotubes/carbon fiber composites synthesized directly and rapidly via an innovative induction heating technique, Carbon 175 (2021) 101–111.

[9] T. Wang, Y. Zhang, G. Bai, X. Liu, Constructing FeCoNiCrAl high entropy alloys with tunable nanograin size and crystal structure to boost polarization loss for enhanced microwave absorption performances, J. Mater. Res. 39 (2024) 248–261.

[10] Biconical prisms Ni@C composites derived from metal-organic frameworks with an enhanced electromagnetic wave absorption, Carbon 184 (2021) 115–126.

[11] Z. Liu, Y. Zeng, L. Long, Y. Li, W. Zhou, Lightweight UiO-66 MOF/CNT composites achieving high electromagnetic wave absorption, Diam. Relat. Mater. 157 (2025) 112500.

[12] B. Liu, B. Liang, J. Xiao, M. Feng, H. Cheng, Y. Li, Y. Cheng, K. Zhang, MOF derived NiFe@C composites with controllable multi-dimensional microstructures for broadband microwave absorption, Compos. Part Appl. Sci. Manuf. 176 (2024) 107869.

[13] Y. Fang, C. Dong, W. Wang, X. Liang, M. Fang, Y. Zhang, R. Han, Broadband Microwave-Absorbing GO-CNT Nanocomposites Enabled by Synergistic FeCoNi Ternary Catalysis, Nanoscale (2025).

[14] Y. Duan, H. Pang, H. Zhang, Structure and composition design on ternary CNT@$ZnFe_2O_4$@ZnO composite utilized as enhanced microwave absorbing materials, Diam. Relat. Mater. 120 (2021) 108701.
